# Supplementary material for: Spatial variability of and effect of light on the cœlenteron pH of a reef coral
Source: Commun Biol. 2024 Feb 29;7:246. doi: 10.1038/s42003-024-05938-8 (PMC10904758; doi:10.1038/s42003-024-05938-8)
Supplement: Supplementary file 2 — Supplementary Material [file 42003_2024_5938_MOESM2_ESM.pdf]

Spatial variability of and effect of light on the cœlenteron pH of a reef coral

Lucas Crovetto[1,2], Alexander Venn[1], Duygu Sevilgen[1], Sylvie Tambutté[1], Eric Tambutté[1]

[1] Centre Scientifique de Monaco, Département de Biologie Marine, 98000 Monaco

[2] Sorbonne Université – ED 515 Complexité du Vivant, 75005 Paris

Supplementary materials

Supplementary Table 1: pH values obtained in this study for seawater and the cœlenteron (HDD and LDD) of *S. pistillata* in the light (200 µmol photons.m^{-2}.s^{-1}) and the dark. Values are mean ± SD. All *P-value* are < 0.05 meaning that all $pH_{cœl}$ measured are significantly different from pH_{SW}.

	**pH (NBS)**	**ΔpH to external seawater**	***P-value* for comparison to external seawater**
**Seawater**	8.08 ± 0.04		
**Cœlenteron (HDD)**			
Light	8.80 ± 0.13	0.72	< 0.05
Dark	7.63 ± 0.23	0.45	< 0.05
**Cœlenteron (LDD)**			
Light	7.67 ± 0.14	0.41	< 0.05
Dark	7.69 ± 0.09	0.39	< 0.05

Supplementary Table 2: **Results of t-test for comparison of $pH_{cœl}$ between polyps and cœnosarc in HDD tissues under light conditions (Figure 2c), Spearman correlation between $pH_{cœl}$ in polyps and cœnosarc and light intensity in HDD tissues (Figure 3) and two way ANOVA for comparison of $pH_{cœl}$ between HDD and LDD tissues under light and dark conditions (Figure 4).** HDD = high dinoflagellate density; LDD = low dinoflagellate density.

**Variable**	**Effect**	***df***	***Statistical value***	***P-value***	***Post hoc***
$pH_{cœl}$ (t-test)	Polyp/cœnosarc	3	t = 0.20365	> 0.05	
$pH_{cœl}$ Polyp (Spearman correlation)	Light intensity		S = 16.598, rho = 0.80	< 0.05	
$pH_{cœl}$ Cœnosarc (Spearman correlation)	Light intensity		S = 6, rho = 0.93	< 0.05	
$pH_{cœl}$ (two-way ANOVA)	Area (HDD/LDD)	1	F = 202.04	< 0.05	HDD > LDD
	Light/Dark	1	F = 393.84	< 0.05	Light > Dark
	Area*L/D	1	F = 186.02	< 0.05	HDD light > HDD dark and LDD light/dark

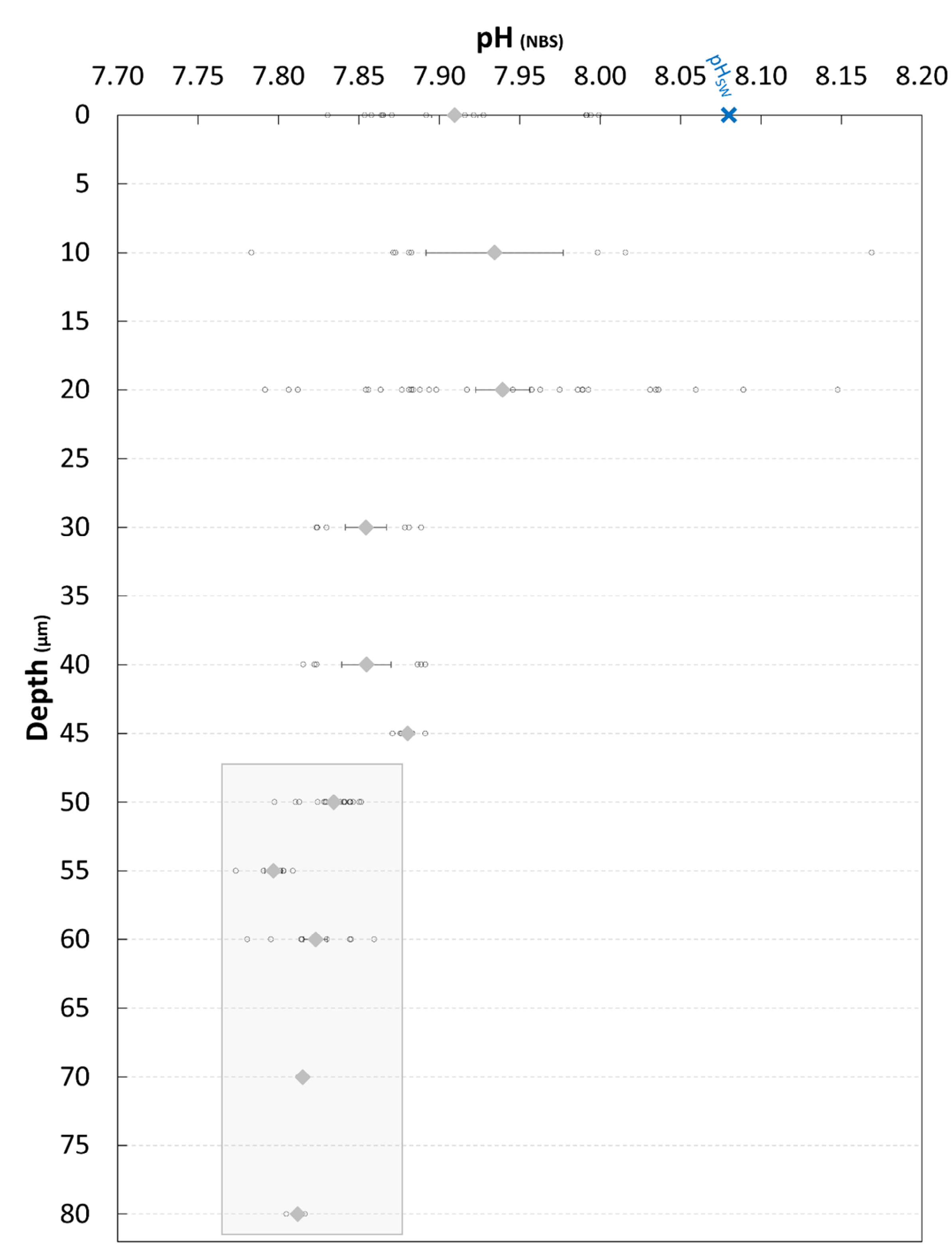

Supplementary Figure 1: pH depth profile with a LIX microsensor in the cœnosarc (tissue with a low dinoflagellate density) of a *S. pistillata* microcolony in the light. Representative pH depth profile recorded on the cœnosarc with the sensor tip inserted into the tissue considered as reference depth 0; depths represent the

interior of the cœnosarc until reaching the maximum depth (80 µm); values are mean ± SE and individual data points are shown. Light grey rectangle represents depths at which the microsensor is inside the cœlenteron.

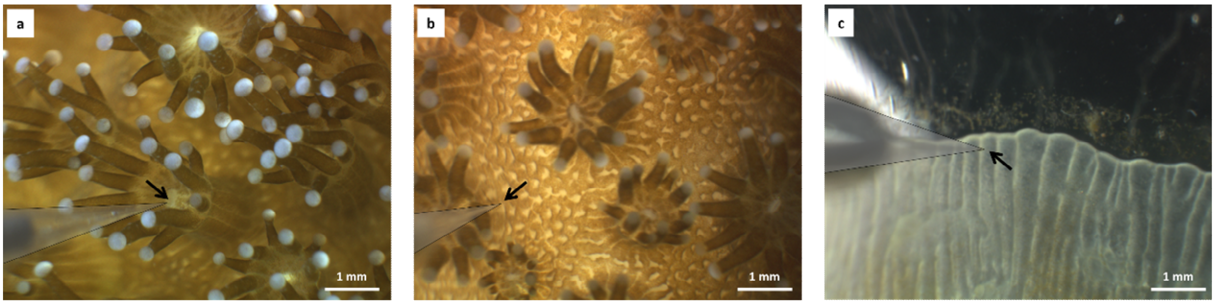

Supplementary Figure 2: Macroscope images of the insertion of a microsensor in a *S. pistillata* microcolony. (**a**) Insertion of a microsensor in a polyp of HDD tissues. (**b**) Insertion of a microsensor in the cœnosarc of HDD tissues. (**c**) Insertion of a microsensor in the cœnosarc of LDD tissues. For a better understanding of the images, the shape of microsensors is highlighted in light grey triangle. Black arrows show the zones where the microsensor is inserted for pH measurements.
