# Supplementary material for: Spatial variability of and effect of light on the cœlenteron pH of a reef coral
Source: Commun Biol. 2024 Feb 29;7:246. doi: 10.1038/s42003-024-05938-8 (PMC10904758; doi:10.1038/s42003-024-05938-8)
Supplement: Supplementary file 3 — Description of Additional Supplementary Files [file 42003_2024_5938_MOESM3_ESM.pdf]

Description of Additional Supplementary Files

File name: Supplementary Data
Description: Values for all data points of all the main figures.
